# Identifying Success Factors for Optimizing COVID-19 Vaccine Uptake Among Indigenous Populations in Taiwan: Cross-Sectional Questionnaire Survey

**DOI:** 10.2196/75278

**Published:** 2025-09-10

**Authors:** Jia-Chi Tu Abùs Isbabanal, Ray-E Chang, Liam O'Neill, Ting-Hung Chen, Po-Han Lee, Tsan-Teng Ou

**Affiliations:** 1 Namasia District Public Health Center, Department of Health, Kaohsiung City Government, Kaohsiung, Taiwan; 2 Department of Family Medicine, Saint Paul's Hospital, Taoyuan, Taiwan; 3 Institute of Health Policy and Management, College of Public Health, National Taiwan University, No. 17, Xu-Zhou Rd., Rm. 639, Zhongzheng District, Taipei, 10055, Taiwan, 886 233668069; 4 Department of Rehabilitation and Health Services, College of Health and Public Service, University of North Texas, Denton, TX, United States; 5 Center of Indigenous Health Care, Department of Community Health, Kaohsiung Medical University Chung-Ho Memorial Hospital, Kaohsiung, Taiwan

**Keywords:** COVID-19, indigenous, health belief model, vaccine hesitancy, health inequity

## Abstract

**Background:**

The COVID-19 pandemic has devastated economies and strained health care systems worldwide. Vaccination is crucial for outbreak control, but disparities persist between and within countries. In Taiwan, certain indigenous regions show lower vaccination rates, prompting comprehensive inquiries.

**Objective:**

This study aims to identify predictors for COVID-19 vaccination and develop strategies for indigenous communities.

**Methods:**

This cross-sectional study, conducted from May 13 to July 18, 2022, surveyed indigenous community members older than 55 years residing in a mountain area in southern Taiwan. Based on the health belief model, the questionnaire covered sociodemographic factors, health-related issues, and trust in physicians. The analysis included bivariate analysis, logistic regression, and mediation analysis.

**Results:**

Most participants (N=203) were aged 55‐64 years (102/203, 50.2%), female (129/203, 63.5%), married (104/203, 51.2%), with low education (165/203, 81.3%), and engaged in agriculture (79/203, 38.9%) or were unemployed (104/203, 51.2%). Logistic regression revealed that unvaccinated individuals were significantly more likely to perceive lower COVID-19 threats (*P*=.03), fewer vaccination benefits (*P*=.04), higher barriers to vaccination (*P*=.02), and weaker responses to external cues to action (*P*<.001), while no significant differences were observed in trust in physicians. Mediation analyses further indicated that trust in physicians influenced vaccine uptake indirectly through perceived barriers. The indirect effect was statistically significant (95% bootstrap CI 0.013 to 0.437), suggesting a full mediation effect.

**Conclusions:**

Effective pandemic prevention strategies for indigenous communities should be grounded in a nuanced understanding of local needs and incorporate bottom-up approaches to avoid cultural saturation and the exacerbation of existing health disparities. Ensuring the accuracy and clarity of vaccine-related information received by indigenous older adults is essential. Local health authorities should consider deploying health care professionals to engage directly with indigenous older adults and their caregivers, delivering culturally appropriate and evidence-based information to address concerns regarding vaccine safety and perceived risks. Such efforts are critical to strengthening vaccine confidence and increasing vaccination uptake in these communities.

## Introduction

A growing body of research has focused on the impact of the COVID-19 pandemic on aboriginal and indigenous populations throughout the world. Much of this research has explored ways to increase vaccination rates and access to health services among indigenous populations in various countries, such as Australia, Brazil, Guatemala, India, and the United States [1-5]. Globally, due to a legacy of systemic racism and marginalization, indigenous peoples have a greater burden of disease than nonindigenous peoples. For example, indigenous peoples have had higher rates of cardiovascular disease, diabetes, and infectious diseases, and this has resulted in a lower life expectancy [6]. In terms of social determinants, their health is impacted by numerous chronic problems, such as lower educational attainment, geographic isolation, lack of access to culturally appropriate health care, and persistent poverty.

In the absence of a coordinated program that is targeted toward indigenous peoples, they are likely to have higher rates of infection, serious complications, and mortality due to COVID-19 [2,5,7]. Vaccination is the most effective strategy to protect the population from adverse outcomes due to the pandemic. However, indigenous peoples face numerous barriers to getting vaccinated. These may include: geographic isolation, lack of access to health care, cultural barriers (such as language), and vaccine hesitancy [6].

Taiwan’s indigenous populations are Austronesian people who are officially recognized as 16 distinct ethnic groups. They comprise approximately 569,000 indigenous people, accounting for about 2% of the national population. In 2021, Taiwan’s Ministry of Health and Welfare launched a nationwide COVID-19 vaccination program aimed at optimizing vaccine uptake, including targeted efforts for the indigenous population. As part of the vaccine rollout, priority groups were identified based on risk profile. Notably, indigenous persons aged 55‐64 years were granted early access to vaccination compared with their nonindigenous counterparts in the same age cohort. To promote vaccination uptake, the government implemented a broad range of outreach strategies, including mass media campaigns, social media promotion, and incentive-based approaches such as the distribution of household goods or gift vouchers. Health education materials, such as informational videos and visual aids, were disseminated in multiple languages, with Mandarin and Taiwanese serving as the primary languages, to enhance accessibility and understanding.

The health belief model (HBM) has proven to be an excellent conceptual framework, not only to explore the motives of persons who get vaccinated but also to better understand the reasons for refusing vaccination [8]. The main premise of the HBM model is that existing beliefs and attitudes can predict future behavior. HBM includes 5 major constructs: perceived susceptibility, perceived severity, perceived benefits, perceived barriers, and cues to action. Therefore, this study uses the HBM model and sociodemographic and health-related factors to identify beliefs and attitudes that can predict vaccine uptake among indigenous peoples. The goal is to identify policies and success factors so that public health agencies will be better prepared to mitigate the potential harm to indigenous peoples due to future epidemics.

This article describes the planning, implementation, outcomes, and policy implications of this program, as it was experienced by one indigenous community in a remote, mountainous area of Taiwan. We identify the various challenges encountered and how these were overcome through local, bottom-up efforts. The HBM model was used to better understand the underlying factors regarding the decision to accept or reject the COVID-19 vaccine.

This research was conducted in a remote mountain region (253 km²) officially designated as an indigenous region in southern Taiwan. This isolated mountainous region comprises 3 rural villages with a total population of 3143 indigenous residents, the majority of whom belong to the Bunun people (approximately 70%), alongside smaller populations of Kanakanavu and Paiwan peoples. Health care services in the region are limited to a single public health center and a clinic. Residents requiring hospitalization or specialist care must travel up to 2 hours to reach the nearest regional referral hospital. To promote COVID-19 vaccination, the local government implements the standard nationwide promotional strategies. However, recognizing language and culture barriers, the public health center independently produced vaccination videos in Kanakanavu, Bunun, and Hla’alua languages, despite the absence of official materials in indigenous languages from the central government. These videos were disseminated through “Line,” Taiwan’s most widely used social media platform, significantly enhancing public awareness and understanding of COVID-19 and the importance of vaccination within the community.

Most previous studies relied on vaccination intention as a proxy for actual vaccine uptake [9]. However, there may be a discrepancy between what people intend to do and what they actually do, namely, receive the vaccine. For example, Harris et al [10] found that only about half of ‘‘intending’’ recipients of seasonal influenza vaccination actually got vaccinated. Intention to vaccinate may change over time and may be subject to societal norms, such as a marketing campaign to promote vaccinations [11,12]. To address this gap, this study uses actual vaccine uptake rather than vaccine intention. Survey responses were validated by checking the participants’ vaccine status with a national database of immunization records.

## Methods

This study adhered to the Strengthening the Reporting of Observational Studies in Epidemiology (STROBE) guidelines for cross-sectional research (Checklist 1).

### Study Design and Participants

This study is an observational, cross-sectional interview design. Grounded in the HBM framework, the study framework incorporated sociodemographic characteristics, health-related factors, and trust in physicians. The primary outcome of interest was actual COVID-19 vaccine uptake, as verified through the national immunization registry. Explanatory variables included: (1) sociodemographic and health-related factors; (2) perceived threats; (3) perceived benefits; (4) perceived barriers; (5) cues to action, defined as external prompts encouraging vaccination; and (6) trust in physicians. Previous research has demonstrated that trust in health care providers can influence vaccination intention or behavior both directly and indirectly through health belief constructs [13,14]. Accordingly, this study also hypothesizes potential pathways from trust in physicians to perceived threats, perceived benefits, and perceived barriers.

Indigenous persons were eligible to be included in the study if they lived in the tribal community for at least 6 months and were 55 years or older, as this group was prioritized to receive the vaccine. (Note that persons younger than 55 years were not eligible to receive the vaccine at this time. Public health measures were developed to address disparities in life expectancy and socioeconomic and sociocultural conditions between indigenous populations and nonindigenous populations. Therefore, indigenous people who are 55 years and older are eligible to receive all the rights and benefits granted to nonindigenous persons who are 65 years and older in Taiwan. Persons who could not understand the survey questions were excluded, such as those with a mental disorder or other conditions with whom the team did not have the ability to communicate.

Participants were recruited from an indigenous region in southern Taiwan through local health stations, clinics, churches, community offices, and health events. Researchers initially consulted participants verbally regarding their willingness to participate, followed by an on-site explanation of the study and the collection of written informed consent. Data were collected using a structured, face-to-face questionnaire administered by trained interviewers, with each session lasting approximately 20 minutes. Although the majority of interviews were conducted in person, some were carried out by telephone due to infection control measures during the COVID-19 pandemic or based on participants’ preferences. When necessary, certified interpreters were available to assist participants who required support in understanding the questionnaire in their indigenous languages, ensuring accurate comprehension. In addition to the formal survey, researchers also conducted informal follow-up conversations to gain deeper insights into participants’ reasons for vaccine hesitancy or nonuptake.

### Survey Instrument

In this study, the survey instrument of the HBM (including constructs such as perceived threat, benefits, barriers, and cause to action) was primarily adapted from the questionnaire developed by Huang et al [15], which focused on influenza vaccination among Taiwanese individuals. To contextualize the instrument for COVID-19, additional modifications were made with reference to studies by Hossain et al [16], Walrave et al [17], and Shmueli [18]. Items measuring trust in physicians were informed by Hall et al [19] and subsequently adapted to align with the objectives of this study. To enhance content validity and cultural appropriateness, the survey was reviewed and revised by 2 public health experts, as well as 2 attending physicians with over 20 years of experience delivering frontline medical care in indigenous communities. Their feedback helped refine the wording to improve clarity and comprehension for study participants.

The survey instrument consisted of a total of 40 questions structured into 6 sections. The first section, sociodemographic and health-related factors, included the following items: age, gender, marital status, educational level, occupation, monthly income, welfare status, chronic disease or major injury, obesity, tobacco use, alcohol use, flu vaccination during the past year, and self-reported health status. The survey also included items corresponding to the following constructs: (1) perceived threats, including susceptibility and severity; (2) perceived benefits; (3) perceived barriers; (4) cues to action; (5) trust in physicians; and (6) actions to receive the COVID-19 vaccine. The specific survey items are presented in Textbox 1. The construct “Actions to Receive the COVID-19 Vaccine” was measured as a binary variable, indicating whether the participant had received at least one dose of the vaccine (0=unvaccinated; 1=at least one dose). All other constructs were assessed using a 5-point Likert Scale, ranging from 1 (“strongly disagree”) to 5 (“strongly agree”), to evaluate participants’ beliefs and attitudes.

Textbox 1. **Items of the health belief model and physician trust constructs.**

**Perceived Threats**

1. I am worried about the likelihood of getting infected by COVID-19.
2. I am at high risk of COVID-19 because of my health conditions.
3. If I were infected with the COVID-19 virus, it would have important health consequences for me.
4. If I were infected with the COVID-19 virus, my health would be severely affected.
5. If I were infected with the COVID-19 virus, my health would be significantly reduced.

**Perceived Benefits**

1. I think vaccination is good because it will make me less worried about COVID-19.
2. I believe vaccination will decrease my risk of getting infected with COVID-19.
3. I think the complications of COVID-19 will decrease if I get vaccinated and then get infected with the coronavirus.

**Perceived Barriers**

1. I am worried that the possible side effects of the COVID-19 vaccination would interfere with my usual activities.
2. I am concerned about the efficacy of the COVID-19 vaccine.
3. I have a concern that I may receive a faulty/fake COVID-19 vaccine.
4. It concerns me that the development of a COVID-19 vaccine is too rushed to test its safety properly.
5. I am concerned about the long-term side effects of the COVID-19 vaccination.

**Cues To Action**

1. The chances of me getting vaccinated against COVID-19 will increase if opinion leaders on social media express support for the benefits of the vaccine.
2. The chances of me getting vaccinated against COVID-19 will increase if friends and family express support for the benefit of the vaccine.
3. If my workplace takes care of vaccinating the workers against COVID-19, I will get vaccinated.
4. The chances of me getting vaccinated against COVID-19 will increase if workers in the field of religion (such as priests, nuns, etc) express support for the benefit of the vaccine.
5. The chances of me getting vaccinated against COVID-19 will increase if local ethnic opinion leaders (such as tribal elders, leaders, respected elders, etc) express support for the benefit of the vaccine.
6. The chances of me getting vaccinated against COVID-19 will increase if local officials (such as village officials, district head, etc) express support for the benefit of the vaccine.
7. Rewards (such as gifts or vouchers) for vaccinating against COVID-19 will increase my willingness to get vaccinated against COVID-19.

**Trust in Physicians**

1. You completely trust public health physicians’ decisions about which medical treatments are best.
2. Public health physicians are totally honest in telling their patients about all of the treatment options available for their conditions.
3. Public health physicians always use their very best skills and effort on behalf of their patients.
4. All in all, I trust public health physicians completely.

**Actions to receive the COVID-19 vaccine**

1. I had received at least one dose of the COVID-19 vaccine (Yes/No)

### Validity and Reliability

To evaluate the instrument’s validity, expert review and exploratory factor analysis (EFA) were used. EFA was conducted using principal axis factoring to explore the underlying factor structure of the questionnaire items. Given the potential correlation among factors, direct oblimin rotation was applied. Sampling adequacy was confirmed by the Kaiser-Meyer-Olkin measure and Bartlett test of sphericity. The Kaiser-Meyer-Olkin value was 0.82, indicating excellent sampling adequacy, and the Bartlett test was significant (*χ*²_253_=4,624; *P*<.001), supporting the factorability of the correlation matrix. Five components were extracted from 23 items, explaining a total of 79.43% of the variance. After oblique rotation, the variance was distributed across the following components. The first component, cause to action, consisted of 6 items and accounted for 27.48% of the total variance. The second component, perceived threats, comprised 5 items and explained 17.27% of the variance. The third component, Trust in Physicians, comprised 4 items and accounted for 18.82% of the variance. The fourth component, perceived barriers, included 5 items and explained 18.16% of the variance. The fifth component, perceived benefits, consisted of 3 items and accounted for 18.28% of the variance. All items demonstrated better factor loadings and converged into 5 dimensions as expected. Reliability was evaluated using Cronbach α. Cronbach α was used to verify internal consistency. All subscales demonstrated high internal consistency, with α coefficients exceeding 0.84.

### Statistical Analysis

The questionnaire data were imported into SPSS software (version 25; IBM Corp) and identified by codes. Reliability and validity were examined using Cronbach α and EFA. To characterize the study population, descriptive analytics were revealed by frequency, percentage, mean, and SD. Bivariate analysis, including Pearson chi-square test and independent sample *t* tests, was used to analyze the correlation between dependent and independent variables. Finally, multivariate binary logistic regression was performed in order to investigate determinants of COVID-19 vaccination; only variables found to be significantly (*P*<.05) associated with COVID-19 vaccination in bivariate analysis were included in the regression. Mediation analysis was performed using PROCESS version 4.2 for SPSS [20], using model 4 to examine the indirect effect of perceived threat, perceived benefits, and perceived barriers on the association between trust in physicians and COVID-19 vaccine uptake. Bootstrapping with 5000 resamples was used to estimate the 95% CIs for both the direct and indirect effects. An indirect effect was considered statistically significant if the CI did not include 0.

### Ethical Considerations

All procedures performed in studies involving human participants were in accordance with the ethical standards of the Institutional Review Board of the Kaohsiung Medical University Chung-Ho Memorial Hospital (serial number: KMUHIRB-SV(I)-20220004) and with the 1964 Helsinki declaration and its later amendments or comparable ethical standards. Out of serious ethical considerations, we also convened consultation meetings about cultural risk assessment by the Council of Indigenous Peoples and received endorsement and approval of the community assembly in the local community (serial number: CRB-111‐005). Participants were recruited in person by the research team, who provided an oral explanation of the study’s objectives and procedures. Written informed consent was obtained from each participant prior to the commencement of the survey. All data collected in this study were anonymized and securely stored, with no personally identifiable information retained, to ensure participant confidentiality.

## Results

### Participants

Table 1 provides descriptive characteristics of the indigenous participants. A total of 203 participants completed the interview. The majority of those included were aged around 55‐64 years (102/203, 50.2%), female (129/203, 63.5%), married (104/203, 51.2%), and had an educational level of junior high school or below (165/203, 81.3%). Most of the participants were unemployed (104/203, 51.2%) or engaged in agricultural work (79/203, 38.9%), with monthly available money below NT $9999 (average exchange rate at the time of study was US $1=NT $29.75) (154/203, 75.9%), much less than the minimum wage of the same year ( NT $25,250). Most of the participants had chronic diseases (173/203, 85.2%), and about half of the participants had not been vaccinated against influenza in the past year (104/203, 51.2%). Persons with at least a high school education were more likely to get vaccinated than those who did not complete high school (97.4% vs 81.8%; *P*=.02). Persons who abstained from alcohol were more likely to get vaccinated than those who drank alcohol at least once a month (89.8% vs 78.9%; *P*=.03).

**Table 1.** Descriptive characteristics of indigenous participants (N=203).

Demographic	Received one or more doses of the COVID-19 vaccine			
	No (n=31)	Yes (n=172)	Total (N=203)	*P* value
Age (years), n (%)				.83^a^
55‐64	17 (54.8)	85 (49.4)	102 (50.2)	
65‐74	10 (32.3)	65 (37.8)	75 (36.9)	
75 and older	4 (12.9)	22 (12.8)	26 (12.8)	
Sex, n (%)				.78^a^
Male	12 (38.7)	62 (36.0)	74 (36.5)	
Female	19 (61.3)	110 (64.0)	129 (63.5)	
Marriage, n (%)				.002^a^^b^
Unmarried (including divorced and widowed)	23 (74.2)	76 (44.2)	99 (48.8)	
Married	8 (25.8)	96 (55.8)	104 (51.2)	
Education level, n (%)				.02^a^^b^
Junior high school or below	30 (96.8)	135 (78.5)	165 (81.3)	
High school/high vocational school or above	1 (3.2)	37 (21.5)	38 (18.7)	
Occupation, n (%)				.64^a^
Part-time job	0 (0.0)	7 (4.1)	7 (3.4)	
Public servant	0 (0.0)	1 (0.6)	1 (0.5)	
Agriculture-related	13 (41.9)	66 (38.4)	79 (38.9)	
Business-related	0 (0.0)	5 (2.9)	5 (2.5)	
None	16 (51.6)	88 (51.2)	104 (51.2)	
Others	2 (6.5)	5 (2.9)	7 (3.4)	
Disposable income (NT $)^c^, n (%)				.26^a^
0‐9999	26 (83.9)	128 (74.4)	154 (75.9)	
10,000 and above	5 (16.1)	44 (25.6)	49 (24.1)	
Welfare status, n (%)				.17^a^
No	31 (100.0)	162 (94.2)	193 (95.1)	
Yes	0 (0.0)	10 (5.8)	10 (4.9)	
Living status, n (%)				.06^a^
Living alone	10 (32.3)	31 (18.0)	41 (20.2)	
Living with relatives	21 (67.7)	141 (82.0)	162 (79.8)	
Chronic disease, n (%)				.39^a^
No	3 (9.7)	27 (15.7)	30 (14.8)	
Yes	28 (90.3)	145 (84.3)	173 (85.2)	
Diagnosis of serious injury or illness, n (%)				.50^a^
No	25 (80.6)	129 (75.0)	154 (75.9)	
Yes	6 (19.4)	43 (25.0)	49 (24.1)	
Obesity, n (%)				.29^a^
No	22 (71.0)	105 (61.0)	127 (62.6)	
Yes	9 (29.0)	67 (39.0)	76 (37.4)	
Smoking, n (%)				.11^a^
No	20 (64.5)	134 (77.9)	154 (75.9)	
Yes	11 (35.5)	38 (22.1)	49 (24.1)	
Alcohol consumption, n (%)				.03^a^^b^
No	11 (35.5)	97 (56.4)	108 (53.2)	
Yes	20 (64.5)	75 (43.6)	95 (46.8)	
The habit of chewing betel nut, n (%)				.03^a^^b^
No	24 (77.4)	156 (90.7)	180 (88.7)	
Yes	7 (22.6)	16 (9.3)	23 (11.3)	
Influenza vaccination in the past year, n (%)				.005^a^^b^
No	23 (74.2)	81 (47.1)	104 (51.2)	
Yes	8 (25.8)	91 (52.9)	99 (48.8)	
Self-reported health status, mean (SD)	63.23 (11.73)	61.51 (15.28)		.46^d^
Perceived threat, mean (SD)	3.42 (1.04)	3.86 (1.05)		.02^b^^d^
Perceived benefit, mean (SD)	2.80 (1.25)	4.11 (0.89)		<.001^b^^d^
Perceived barrier, mean (SD)	1.93 (0.82)	2.76 (1.23)		<.001^b^^d^
Cues to action, mean (SD)	2.07 (1.11)	3.47 (1.21)		<.001^b^^d^

a Chi-square test.

b Statistically significant values.

c Average exchange rate at time of study was US $ 1 = NT $29.67.

d Independent samples *t* test.

### Factors Associated With Action to COVID-19 Vaccination

Logistic regression analysis without the HBM scale (Table 2, Model 1) included sociodemographic and health-related factors and trust in physicians, and the stepwise backward conditional method was used to select the important variables and fit the model. The results show that marriage status (adjusted odds ratio [AOR] 3.931, 95% CI 1.602 to 9.648; *P*=.003), influenza vaccination in the past year (AOR 3.188, 95% CI 1.291 to 7.872; *P*=.01), and trust in physicians (AOR 1.452, 95% CI 1.004 to 2.099; *P*=.047) are significantly positively correlated with the COVID-19 vaccination.

When the constructs of HBM were added to the model (Table 2, model 2), marriage status (AOR 4.063, 95% CI 1.384 to 11.926; *P*=.01), influenza vaccination in the past year (AOR 3.367, 95% CI 1.063 to 10.662; *P*=.04), perceived threat (AOR=1.922, 95% CI 1.053 to 3.507; *P*=.03), perceived benefits (AOR 1.723, 95% CI 1.023 to 2.901; *P*=.04), perceived barriers (AOR 0.412, 95% CI 0.199 to 0.855; *P*=.02) and cues to action (AOR 2.603, 95% CI 1.461 to 4.637; *P*<.001) were significantly positively correlated to the COVID-19 vaccine; however, trust in physicians was not selected into model 2.

**Table 2.** Factors associated with actions to receive the COVID-19 vaccine among indigenous participants by backward stepwise multivariate binary logistic regression (n=203).

	Model 1^a^				Model 2^b^			
	Path coefficient	*P* value	95% CI	AOR^c^	Path coefficient	*P* value	95% CI	AOR^c^
Marriage (reference: unmarried [including divorced and widowed])^d^								
Married	1.369	.003^e^	1.602-9.648	3.931	1.402	.01^e^	1.384-11.926	4.063
Education (reference: incomplete high school education)^d^								
Complete high school education	2.060	.05	0.999-61.656	7.850	1.708	.14	0.580-52.561	5.520
Influenza vaccination in the past year (reference: no)^d^								
Yes	1.159	.01^e^	1.291-7.872	3.188	1.214	.04^e^	1.063-10.662	3.367
Trust in physicians health belief model	0.373	.047^e^	1.004-2.099	1.452				
Perceived threat					0.653	.03^e^	1.053-3.507	1.922
Perceived benefits					0.544	.04^e^	1.023-2.901	1.723
Perceived barriers					−0.886	.02^e^	0.199-0.855	0.412
Cues to action					0.957	<.001^e^	1.461-4.637	2.603

a Nagelkerke *R*2=0.230.

b Nagelkerke *R*2=0.535.

c AOR: adjusted odds ratio.

d The reference category in the logistic regression model, against which the odds ratios of other categories are compared.

e Statistically significant values.

### Mediating Effect Between Trust in Physicians and Actions to Receive the COVID-19 Vaccine

The results (Table 3) indicated that trust in physicians was not significantly associated with perceived threat (β=0.013; *P*=.86) or with COVID-19 vaccine uptake (AOR 0.993, 95% CI −0.505 to 0.490; *P*=.98). Given the nonsignificant direct association between trust in physicians and perceived threat, the potential mediating role of perceived threat was not further examined.

The trust in physicians was positively associated with perceived benefits (β=0.188; *P*=.003), and perceived benefits were significantly associated with vaccine uptake (β=0.545; *P*=.04). However, the indirect effect of trust in physicians on vaccine uptake through perceived benefits was 0.104, with a 95% bootstrap CI (−0.005 to 0.334), including 0, indicating no significant mediation effect.

Additionally, trust in physicians was negatively associated with perceived barriers (β=−0.165; *P*=.01), and perceived barriers were significantly associated with vaccine uptake (β=−0.887; *P*=.02). The indirect effect of trust in physicians on vaccine uptake through perceived barriers was 0.143, with a 95% bootstrap CI (0.013 to 0.437), not including 0, indicating a significant full mediation effect.

**Table 3.** Results of the mediating role of perceived threat, benefits, and barriers between trust in physicians and actions to receive the COVID-19 vaccine.

	Path coefficient	*P* value	95% CI^a^ (Bootstrap)
Direct effect			
Trust in Physicians → Perceived Threat	0.013	.86	−0.127 to 0.153
Perceived Threat → Actions to receive the COVID-19 vaccine	0.654	.03^b^	0.052 to 1.255
Trust in Physicians → Perceived Benefits	0.188	.003^b^	0.064 to 0.312
Perceived Benefits → Actions to receive the COVID-19 vaccine	0.545	.04^b^	0.018 to 1.073
Trust in Physicians → Perceived Barriers	−0.165	.01^b^	−0.295 to −0.034
Perceived Barriers → Actions to receive the COVID-19 vaccine	−0.887	.02^b^	−1.619 to −0.154
Trust in Physicians → Actions to receive the COVID-19 vaccine	−0.007	.98	−0.505 to 0.490
Indirect effect			
Trust in Physicians → Perceived Threat → Actions to receive the COVID-19 vaccine	0.008	—^c^	−0.115 to 0.136
Trust in Physicians → Perceived Benefits → Actions to receive the COVID-19 vaccine	0.104	—^c^	−0.005 to 0.334
Trust in Physicians → Perceived Barriers → Actions to receive the COVID-19 vaccine	0.143	—^c^	0.013 to 0.437

a A CI that does not include 0 indicates a statistically significant mediation effect.

b Statistically significant values.

c Not applicable.

## Discussion

### Principal Findings

The findings indicate that marital status, influenza vaccination in the previous year, and trust in physicians were significantly associated with the COVID-19 vaccine uptake before incorporating constructs from the HBM. After adding the HBM constructs, namely perceived threats, perceived benefits, perceived barriers, and cues to actions, marital status, prior influenza vaccination, and all HBM-related factors remained significantly associated with vaccine uptake. However, trust in physicians was no longer a significant predictor after the inclusion of the HBM constructs in the model. To further examine the underlying mechanisms, mediation analyses were performed to assess the potential indirect effects of trust in physicians on vaccine uptake through perceived threats, perceived benefits, and perceived barriers. Results revealed that perceived barriers fully mediated the relationship between trust in physicians and vaccine uptake.

### Trust in Physicians

Before incorporating the HBM constructs into the logistic regression model, trust in the physician was significantly associated with COVID-19 vaccine uptake. However, the association became nonsignificant after the HBM factors were introduced in the model. Subsequent analyses examining the associations between trust in physicians and other HBM constructs revealed significant associations with perceived benefits and perceived barriers, but not with perceived threat. This pattern may reflect the close and longstanding relationships between local indigenous residents and physicians stationed in the community. The study region has extremely limited health care resources, with only 2 clinics serving the entire area. The public health center is the sole facility providing routine vaccination services, and its physicians conduct weekly outreach visits to each village to deliver both primary care and vaccinations. In addition, home visits are arranged for individuals residing in remote areas or with mobility impairments. Given this longstanding physician-community relationship, it is likely that older indigenous adults receive vaccination-related information primarily from physicians.

However, further path analysis indicated that trust in physicians exerted a significant indirect effect on vaccination uptake through perceived barriers, but not through perceived benefits. While previous studies have suggested that trust in health care providers can influence vaccination behavior both directly and indirectly through multiple health belief constructs [21], the present findings indicate that such influence may operate through a more limited pathway, specifically, via perceived barriers.

### Perceived Barriers

In this study, the construct of perceived barriers was significantly negatively associated with COVID-19 vaccine uptake, thus highlighting their detrimental role in vaccination decisions. Barriers included concerns regarding the vaccine development process, doubts about vaccine safety or effectiveness, fear of receiving a defective vaccine, and apprehension about potential severe or long-term side effects. Concerns about vaccine safety have consistently been identified as a major obstacle to vaccination across various populations [22]. Given that COVID-19 is caused by a novel coronavirus first detected in humans, the accelerated development and deployment of its vaccines raised public concerns about insufficient information and unknown long-term effects. Moreover, some vaccine platforms were used in human populations for the first time, contributing to hesitation. Not surprisingly, anxiety surrounding the safety and risks of vaccination, including unverified rumors, was widespread. In this study, worries about the expedited approval process and potential adverse effects were frequently mentioned by participants during informal interviews. These findings are consistent with previous research among an Australian indigenous study [1] and older adults in Taiwan [23], both of which identified safety concerns as central to vaccine hesitancy. Despite the negative influence of perceived barriers, mediation analysis revealed a significant indirect positive effect of trust in physicians on vaccine uptake through perceived barriers. In other words, trust in physicians mitigated the adverse impact of perceived barriers, thereby facilitating vaccine acceptance. This finding highlights the critical role of physician trust in enhancing the credibility and perceived accuracy of vaccine-related information [24], which is particularly important for indigenous older adults, who may face greater structural and informational disadvantages. Overall, these findings underscore the central role of perceived barriers in shaping vaccine behavior and demonstrate that reducing such barriers, partly through fostering trust in physicians, may be essential to improving vaccine uptake in indigenous older adult populations in Taiwan.

### Perceived Threat

The result of logistic regression indicated that respondents who were worried about the epidemic tended to get vaccinated, similar to other studies [25]. The perception of the disease affecting the odds of COVID-19 vaccination has also occurred in Australia, for example. Misconceptions about diseases and vaccines among Aboriginal and Torres Strait Islander people, as well as a lack of cultural sensitivity to Aboriginal health care delivery, contribute to lower vaccination rates [26]. Policies can be used to raise disease awareness among the indigenous population to increase vaccination coverage. In Australia, there are policies for encouraging indigenous people to get vaccinated, such as compiling a vaccination plan for indigenous people, relevant implementation strategies, providing indigenous health education media on the official website of the health unit, including short films and picture cards, and providing culturally sensitive educational materials for companies and institutions that may employ aboriginal people. Indeed, effective risk communication for pandemics is crucial, whereas a lack of trust in the central government and, therefore, a lack of communication strategies about COVID-19 prevention in Indigenous languages was a critical challenge [27,28]. Nevertheless, in contrast to previous research [20], this study found that trust in physicians did not exert an indirect effect on vaccine uptake through perceived threat. This divergence may reflect culturally distinct health worldviews among indigenous older adults. During informal, off-the-record conversations conducted alongside data collection, several unvaccinated participants expressed a fatalistic perspective, with remarks such as, “Life and death are predetermined; vaccines won’t change anything.” Such views suggest a low perceived threat of COVID-19, rooted in a worldview that emphasizes acceptance of natural forces and the cyclical nature of life and death. For some participants, the COVID-19 pandemic was regarded not as a crisis to be feared but rather as part of the broader natural order. This perspective reflects a culturally grounded sense of equilibrium with nature, rather than a deficit in health knowledge or risk awareness.

### Perceived Benefits

The direct effect of perceived benefits on vaccination uptake indicates that individuals who believe they will benefit from receiving the vaccine are more likely to do so, a finding consistent with prior studies [18,29]. In addition, the positive association between trust in physicians and perceived benefits suggests that indigenous adults in this study who reported higher trust in physicians were more likely to recognize the potential advantages of vaccination. This pattern aligns with evidence from other vaccination contexts [30]. However, the indirect effect of trust in physicians on vaccine uptake through perceived benefits was not significant, although it approached significance. Previous research has shown that, in some cases, individuals may perceive the risks of vaccination as outweighing its benefits [31]. Given the prominent role that safety concerns played in this study, it is possible that these concerns attenuated the influence of perceived benefits, thereby limiting the mediating role of this construct. Further research is warranted to explore whether trust in physicians may enhance vaccine uptake through other psychological pathways.

### Cue to Action

This study revealed that the support from opinion leaders in the community for vaccination will increase the chances of indigenous elders being vaccinated. A similar case was observed in American Indian communities and has been advocated by international organizations such as the International Federation of Red Cross and Red Crescent Societies [32-34]. Community opinion leaders include not just tribal opinion leaders and local district chiefs but also religious field workers, friends, and family [35,36]. Informal interviews conducted during this study revealed that several participants expressed sentiments such as “No one around me gets vaccinated,” underscoring a lack of external cues to action that might otherwise prompt vaccination. Therefore, an understanding of the interpersonal relationships and communal networks in the local context is essential for increasing the vaccination rate. Thus, as also identified by studies conducted in various social contexts, cooperating with community opinion leaders requires a bottom-up approach to transforming these leaders into “trusted messengers” in vaccination promotion [37]. Such a strategy, often overlooked by the central government, however, is particularly important for indigenous communities according to our and other studies [38,39].

### Limitations

There are some limitations in this study. First, during the period of data collection, the COVID-19 outbreak happened in this region, so it was challenging to do the interviews without considering compliance with relevant regulations in addition to the methodological restraints and ethical considerations preidentified. In any case, the results of this study are reliable and valid, and it is the first study in Taiwan on indigenous COVID-19 vaccination. Second, since the government has yet to release official data on COVID-19 vaccination rates among indigenous people, relevant information on this matter is based on news reports, the accuracy of which is uncertain. Thus, official statistics are still needed to inform a comprehensive analysis. Third, although the findings may not be generalizable to all indigenous populations, the study’s framework is particularly relevant to regions characterized by indigenous-majority populations, limited health care access, and geographic or transportation barriers. Taiwan comprises 55 officially designated indigenous regions, 30 mountainous and 25 plains regions. The mountainous regions particularly tend to exhibit a strong cultural identity and relative geographic isolation. These conditions are also commonly observed in many indigenous communities around the world. While infrastructure and health care delivery systems may vary across indigenous regions, those facing similar structural constraints are likely to experience comparable challenges in vaccine uptake. Therefore, the proposed framework holds promise for broader applicability within Taiwan and potentially across other national and international indigenous communities.

### Conclusions

This study provides empirical evidence on the determinants of COVID-19 vaccination among indigenous older adults in Taiwan. The findings indicate that key constructs of the HBM, including perceived disease threat, perceived vaccine benefits, concerns regarding vaccine safety and risk, and support from trusted community opinion leaders, are significantly associated with vaccine uptake in this population. Notably, the effect of trust in physicians on vaccination behavior appears to operate indirectly through perceptions of vaccine safety and risk, particularly in the context of a severe and rapidly spreading pandemic involving a novel vaccine. These results underscore the importance of a contextualized and culturally grounded approach to risk and policy communication in indigenous communities. Pandemic prevention strategies should be informed by local values and sociocultural dynamics, with trusted community leaders playing a central role in promoting vaccine acceptance. Vaccine hesitancy in these settings may reflect informed cultural or religious perspectives, rather than misinformation or lack of awareness. Accordingly, a bottom-up approach is recommended, whereby frontline health care professionals engage with community members to understand local beliefs and practices, thereby informing policy development. Furthermore, proactive involvement by community health care providers, through the delivery of clear, culturally sensitive, and trustworthy information to indigenous elders and their caregivers, may help alleviate safety concerns and reduce perceived risks. These efforts may enhance vaccine confidence and ultimately improve vaccination coverage in indigenous regions.

## Supplementary material

10.2196/75278 Checklist 1 STROBE cross-sectional checklist.

## Abbreviations

AOR: adjusted odds ratio
EFA: exploratory factor analysis
HBM: health belief model
STROBE: Strengthening the Reporting of Observational Studies in Epidemiology
